# Pathogenesis and Animal Models of Post-Primary (Bronchogenic) Tuberculosis, A Review

**DOI:** 10.3390/pathogens7010019

**Published:** 2018-02-06

**Authors:** Robert L. Hunter, Jefrey K. Actor, Shen-An Hwang, Arshad Khan, Michael E. Urbanowski, Deepak Kaushal, Chinnaswamy Jagannath

**Affiliations:** 1 Department of Pathology and Laboratory Medicine, University of Texas Health Sciences Center at Houston, Houston, TX 77030, USA; Jeffrey.K.Actor@uth.tmc.edu (J.K.A.); Shen-An.Hwang@uth.tmc.edu (S.-A.H.); Arshad.Khan@uth.tmc.edu (A.K.); Chinnaswamy.Jagannath@uth.tmc.edu (C.J.); 2 Tulane National Primate Research Centre, Covington, LA 70433, USA; michael.urbanowski@jhmi.edu; 3 Center for Tuberculosis Research, The Johns Hopkins University School of Medicine, Baltimore, MD 21231, USA; dkaushal@tulane.edu

**Keywords:** tuberculosis, pathology, pathogenesis, animal model, post-primary, bronchogenic, human, mouse, rabbit, primate

## Abstract

Primary and post-primary tuberculosis (TB) are different diseases caused by the same organism. Primary TB produces systemic immunity. Post-primary TB produces cavities to support massive proliferation of organisms for transmission of infection to new hosts from a person with sufficient immunity to prevent systemic infection. Post-primary, also known as bronchogenic, TB begins in humans as asymptomatic bronchial spread of obstructive lobular pneumonia, not as expanding granulomas. Most lesions regress spontaneously. However, some undergo caseation necrosis that is coughed out through the necrotic bronchi to form cavities. Caseous pneumonia that is not expelled through the bronchi is retained to become the focus of fibrocaseous disease. No animal reproduces this entire process. However, it appears that many mammals utilize similar mechanisms, but fail to coordinate them as do humans. Understanding this makes it possible to use human tuberculous lung sections to guide manipulation of animals to produce models of particular human lesions. For example, slowly progressive and reactivation TB in mice resemble developing human bronchogenic TB. Similarly, bronchogenic TB and cavities resembling those in humans can be induced by bronchial infection of sensitized rabbits. Granulomas in guinea pigs have characteristics of both primary and post primary TB. Mice can be induced to produce a spectrum of human like caseating granulomas. There is evidence that primates can develop bronchogenic TB. We are optimistic that such models developed by coordinated study of human and animal tissues can be used with modern technologies to finally address long-standing questions about host/parasite relationships in TB, and support development of targeted therapeutics and vaccines.

## 1. Introduction

It is widely recognized that gaps in knowledge of susceptibility/resistance to pulmonary tuberculosis (TB) are major impediments to development of new vaccines and host directed therapies [1,2]. For example, how can the immune response be responsible both for protection and massive tissue damage? Why are people with the strongest skin tests immune to primary TB, but at greater risk of severe post-primary TB? Part of the problem is a lack of human tissues. If a paper on the pathogenesis of pulmonary TB is less than 60 years old, then one can be reasonably certain that the author never studied developing tuberculosis in human lung tissue [3]. Such tissues ceased to be available to most investigators with the introduction of antibiotics, rise of modern science and decline in autopsies in the 1950s. Virtually the entire modern literature and the paradigm that guides it are based on animal models of TB [4,5]. However, *Mycobacterium tuberculosis* (MTB) is an obligate human parasite that can only complete its life cycle and be transmitted to new hosts by human lungs. There are many models of the early stages of infection, primary TB, in which the host develops systemic immunity sufficient to control organisms within granulomas. The granulomatous lesions of primary TB are similar in man and animals and are found in both pulmonary and extra pulmonary sites greatly facilitating research. There are also multiple models of latent TB. However, there are few models of post-primary TB, the late stages of disease that occur naturally only in human lungs. Organisms in post-primary lesions evade and subvert otherwise highly effective host responses in local areas of lung to produce most disease and nearly all transmission of infection. Unfortunately, there is much misinformation in the literature. The models of post-primary TB that do exist have not been validated by comparison with corresponding human lesions because investigators have not had access to tissues with such lesions. Nevertheless, human pulmonary lesions were well known and clearly described by pathologists in the pre antibiotic era when such tissues were relatively abundant and continue to be well known by radiologists [6,7,8,9,10,11,12,13,14,15,16,17,18,19].

A number of modern investigators have published excellent papers on the pathology of pulmonary TB and have reviewed the literature from the pre antibiotic era without recognizing the key findings [20,21]. Several problems impeded their work. First, most studies in the past 50 years have examined surgically resected lung tissue, not autopsies. Unfortunately, surgeons can only resect limited disease and patients are almost always treated maximally before surgery. The early lesions of post-primary TB disappear rapidly with therapy and are seldom present in surgical resections [6,15,16,17]. Second, few TB researchers are trained in pathology and even fewer have access to autopsies of patients who died of untreated TB. Finally, before Google Books, the literature on the pathology of post-primary TB from the pre antibiotic era was exceedingly difficult to find. It was also difficult to read because of unfamiliar nomenclature and a paucity of either microscopic or macroscopic images.

Alerted by inconsistencies in the literature, we undertook a decade long search for lung tissues of people with untreated TB, and an extended review of publications from the pre antibiotic era when such tissues were abundant. We also reviewed the modern radiologic literature on pulmonary TB. We located and studied tissues from over 100 cases of untreated pulmonary TB and eventually recognized a new paradigm that TB progresses through three distinct stages of pathology, not just one as is generally believed [22]. The current dominant paradigm views TB as a single stage war-of-attrition between the host and microbe fought in granulomas [4,23]. Both sides employ the host immune response to their benefit. The result somehow depends on the balance between different responses which lead to either protective containment or failure to control organisms. Bronchogenic TB (TB pneumonia) when considered at all, was almost universally attributed to overwhelming of host responses and therefore thought to be of little value in understanding the developing disease [5,24].

**Figure 1:**
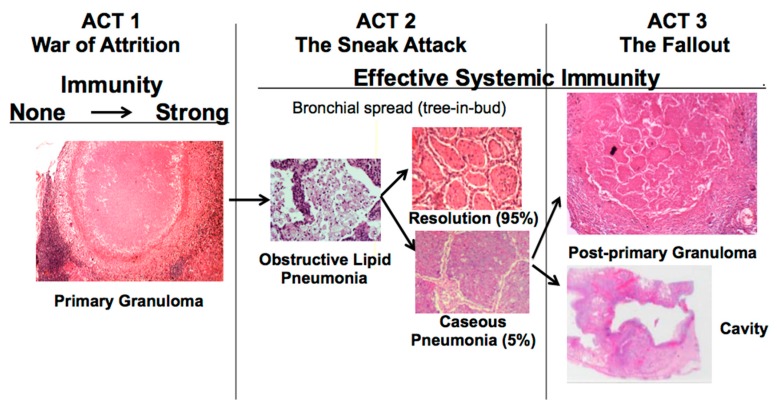
Tuberculosis as a three-act play: A new paradigm for the pathogenesis of pulmonary tuberculosis. The key new component is that post-primary TB begins as a prolonged bronchogenic spread of asymptomatic subclinical infection in alveolar macrophages. Most lesions regress spontaneously. After 1–2 years, a few undergo sudden caseation necrosis to produce cavities and post-primary fibrocaseous disease [22]. Granulomas of post-primary TB are different from those of primary TB in that they contain ghosts of alveolar walls, that are the remnants of caseous pneumonia.

The disease in human lungs is different [22]. The war-of-attrition in granulomas exists only in primary TB. It can produce serious disease in children and immune compromised adults. This stage of disease occurs in most animal models. However, in immune competent humans, the host typically “wins”, and develops immune dependent control in weeks. Post-primary TB (defined as TB that begins in the lung after establishment of systemic immunity stimulated by primary TB) begins asymptomatically as a sneak attack in an otherwise highly immune host, Figure 1. Post-primary TB is also known as bronchogenic TB because it spreads through bronchi rather than either the lymphatics or blood stream. It begins as prolonged, asymptomatic accumulation of mycobacterial antigens and host lipids in alveolar macrophages in preparation for a sudden massive attack that destroys sufficient tissue to produce a cavity capable of supporting large numbers of organisms for transmission to new hosts. Granulomas in post-primary TB are morphologically distinct from those of primary TB, Figure 1. They form late to surround preexisting necrotic caseous pneumonia that fails to be coughed out in cavity formation. Granulomas of primary TB have homogeneous caseum with peripheral lipid. They can be found in all organs. Granulomas of post-primary TB, in contrast, exist only in lungs. They consist of ghosts of alveolar structures surrounded by granulomatous inflammation. Such lesions develop from preexisting caseous pneumonia and have been described so far only in human lungs.

The critical stage that drives post-primary TB, the sneak attack, is asymptomatic and is largely unknown to the scientific community, although it had been described by pathologists for over 125 years and imaged by radiologists for 90 years [6,7,8,9,10,11,12,13,14,15,16,17,18,19]. Post-primary TB begins as subclinical, asymptomatic bronchogenic spread of obstructive lobular pneumonia. Most such lesions regress spontaneously, but some undergo necrosis to produce caseous pneumonia that is either coughed out to leave a cavity or remains to become surrounded by granulomas and fibrocaseous disease. This is not speculation. *The basic concepts displayed in* Figure 1 *are supported by dozens of publications in pathology and radiology written by investigators who personally studied hundreds of cases over a period of nearly 200 years*. In his classic book at the end of this period, Rich wrote “It has been found by all who have studied early human pulmonary lesions that they represent areas of caseous pneumonia rather than nodular tubercles” [15].

**Figure 2:**
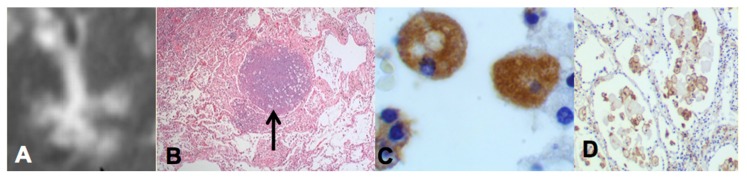
Bronchial obstruction and intracellular accumulation of MTB antigens in developing post-primary TB. The tree-in-bud sign on high resolution CT scan (**A**) is characteristic of developing post-primary TB. It is formed by obstructed bronchioles (tree) and alveoli filled with foamy macrophages (buds). Bronchial obstruction (arrow in **B**) is found in 100% of cases of TB (H&E, 40 magnification). Immunohistochemistry (IHC) stain with rabbit polyclonal antibody against whole killed MTB (**C**,**D**) shows MTB antigens to be intracellular in alveolar macrophages in viable lung tissue. MTB are seldom found in such lesions by either IHC or AFB staining suggesting that very few MTB secrete antigens that are stored for a prolonged period in these cells prior to induction of caseous pneumonia. (**C** 700×, **D** 100× magnification).

Two recent findings contributed to the formation of a new paradigm. First, bronchial obstruction has been found in 100% of cases of pulmonary TB by many pathologists and is the basis of the tree-in-bud sign that is characteristic of advancing TB on CT scans [3,15,19,25] Figure 2A,B. In addition, bronchial obstruction produced by cancer or other disease causes an endogenous lipid pneumonia that resembles developing post-primary TB, including the propensity to undergo caseous like necrosis and cavitation [26,27]. Finally, relief of bronchial obstruction has produced rapid regression of pulmonary TB [15,17,28].

The second finding that contributed to the new paradigm is that secreted, but not somatic, mycobacterial antigens accumulate asymptomatically for months in foamy alveolar macrophages in developing post-primary TB [22], Figure 2C,D. Mustafa et al. reported and we have confirmed that these are secreted, not somatic, antigens of MTB [29]. Such antigens remain intracellular with little or no inflammatory reaction until onset of massive inflammation and caseous necrosis. Scientists have long puzzled over how very few bacilli could produce such sudden massive necrosis in the lung. The finding of secreted mycobacterial antigens slowly and quietly accumulating in foamy alveolar macrophages in the preclinical stage of bronchogenic TB provides a plausible answer. Foamy alveolar macrophages, long thought to be part of non-specific inflammation, are now recognized as major players in the pathogenesis of post-primary TB.

We now know that post-primary TB begins with obstruction of bronchioles that facilitates isolation of parts of the lung for slow, accumulation of mycobacterial antigens and host lipids in alveolar macrophages. The process spreads asymptomatically through bronchi producing a gradual accumulation of mycobacterial antigens and host lipids, ‘munitions’, in preparation for sudden caseous necrosis and expulsion of sufficient lung tissue to produce a cavity capable of supporting proliferation of massive numbers of MTB to be coughed into the environment. As reported by Youmans years ago, “There must be a local breakdown in cellular immunity to infection, which then permits growth of tubercle bacilli to the point where enough tuberculo protein is produced to elicit a local necrotizing allergic reaction.” [30]. We now have the technologies to dissect this process in detail using coordinated studies of animal models and human tissues.

**Animal Models:** Only humans develop the full spectrum of post-primary lesions that result in transmission of MTB. Nevertheless, we believe that it is possible to develop animal models to appropriately mimic nearly the entire spectrum of lesions of human pulmonary TB. Most animals die of a mixture of primary and undeveloped or misaligned post-primary TB. In the absence of informative human tissues, investigators have not been able to validate animal models. The idea that cavities develop as granulomas that erode into bronchi was advanced by work on *M. bovis* in rabbits without recognition of the differences between infections caused by *M. bovis* and MTB [5,16,31]. *M. bovis* causes an exaggerated and prolonged primary TB, but does not produce post-primary TB with bronchogenic spread. Studies in guinea pigs are regularly justified by the statement that they produce ‘human like granulomas’ [24]. That is true, but primary granulomas represent only one type of human lesion. Mice are criticized because they do not produce caseating granulomas without recognition of the fact that their lesions resemble other phases of human TB [32]. Non-human primates are considered to be essential models for both scientific and regulatory reasons, but we have found no direct comparisons with human pulmonary pathology [33]. These statements all manifest lack of understanding of the pathology of the characteristic lesions of human TB.

**Figure 3:**
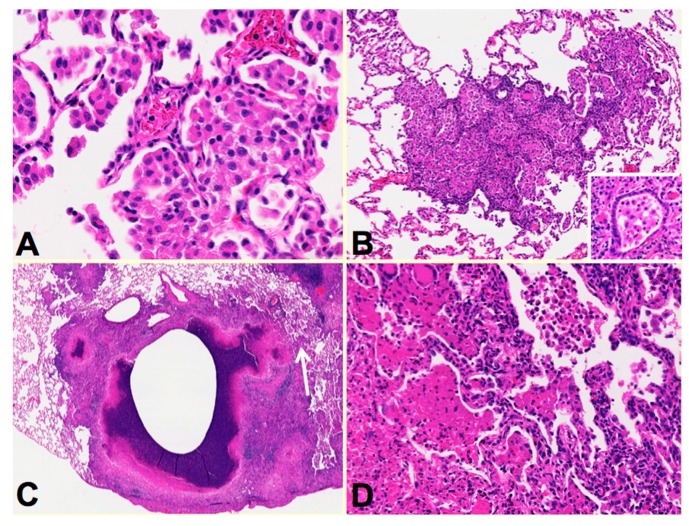
Multiple stages of post-primary TB in a single rabbit lung. Sensitized rabbits infected with MTB via bronchoscopy develop multiple lesions that resemble various stages of the human disease. The earliest stage (**A**) is collection of alveolar macrophages in focal areas of alveoli. Next, (**B**) the alveolar walls become thickened with lymphocytes and the macrophages become variably foamy. Bronchial obstruction (**B** insert) is associated with these lesions. Larger masses of the pneumonia undergo caseation necrosis and soften to produce cavities (**C**). The caseation necrosis may expand in some areas (**C** arrow). A higher magnification of this area (**D**) shows developing caseous pneumonia adjacent to viable alveoli containing macrophages. (all H&E **A** 400×, **B** 200×, **C** 40× and **D** 400×).

**Rabbits:** The availability of human tissues and modern technologies introduces new possibilities. Rabbits are a particularly good example. As advocated by Medlar who spent 15 years studying TB in rabbits and humans, one can produce lesions that resemble particular human lesions by producing the conditions in animals that occur at specific points of the disease in humans [16]. In particular, he developed a rabbit model of bronchogenic caseous pneumonia in rabbits. This model has been further developed by Kubler et al. who produced a model of cavities in rabbits [34], Figure 3. Rabbis were sensitized with multiple injections of heat-killed *M. bovis* in incomplete Freund’s adjuvant followed by installation of viable MTB directly in to the lung by bronchoscopy to produce post-primary disease. The lungs were examined around two months after infection when the animals showed signs of clinical disease. Individual rabbit lungs showed multiple stages of disease in a single lung as is typical of human post-primary TB. Each of the stages resembled the one of the lesions observed in developing human disease. The earliest lesions were collections of macrophages in alveolar spaces, Figure 3A. The alveolar walls then became thickened with lymphoid cells, Figure 3B. These lesions, especially the larger ones, typically are associated with bronchial obstruction that is evidence of bronchogenic spread of infection. By close examination, larger lesions were composed of masses of necrotic caseous pneumonia some of which had central cavities, Figure 3C. Granulomatous tissue (epitheloid cells and fibroblasts surrounded by lymphocytes) was present around the older, but not younger appearing lesions. This is consistent with the observation that granulomas in human post-primary TB form only in response to existing caseous pneumonia [3,7,22]. In some areas, foamy alveolar macrophages were present in alveoli adjacent to the caseous masses. Some of these had undergone necrosis expanding the caseous mass, Figure 3C arrow. Figure 3D is a higher power image of this region showing the juxtaposition of developing caseous pneumonia and lobular pneumonia. These changes are all similar to the pathology of developing post-primary TB in humans where cavities develop from dissolution of caseous pneumonia and post-primary granulomas form only secondarily in response to necrotic caseous pneumonia that is not coughed out in cavity formation. 

**Figure 4:**
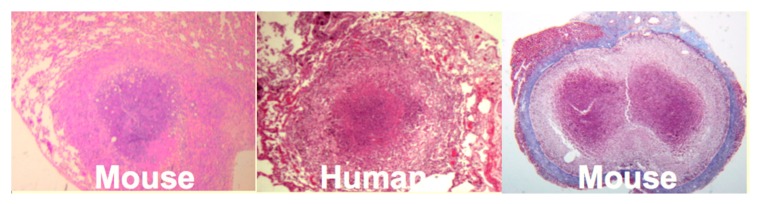
Caseating Granulomas in Humans and Mice (35). C57Bl/6 mice produce classic pulmonary caseating granulomas following injection of a high dose of MTB in oil into the lungs of a sensitized animals (LEFT). Human caseating granuloma for comparison (MIDDLE). Mice injected i.v. with MTB one day after i.p injection of TDM in oil produce caseating granulomas with a fibrous capsule in the peritoneal cavity. (RIGHT, Trichrome connective tissue stain). This lesion contained many MTB after most had been eliminated from other parts of the body. Rupture of the capsule of this type of lesion was associated with reactivation TB in the lungs (Photos at 20× Mag).

**Mice:** Mice have been widely criticized as a model of TB because they do not produce caseating granulomas following low dose aerosol infection. However, by replicating the observation that human caseating granulomas occur when large numbers of organisms are localized in lipid rich tissue of sensitized people, we produced classic caseating granulomas in mice [35], Figure 4. Two mechanisms of necrosis were identified in such lesions. The first was a T cell reaction specific for trehalose 6,6′ dimycolate (TDM). The second appeared to be infarction produced by vascular occlusion as observed in DTH [36]. Modification of the protocols of prior immunization, dose, route and vehicle of challenge infection produced a series of caseating granulomas, each of which resembled a particular human lesion. Most animal models of TB simply monitor lesions as they develop after aerosol infection in order to reproduce the route of infection. Since the animals are unnatural hosts, this des not reproduce many events that take place months or years later in the natural hosts; humans. The models we described can be experimentally manipulated to address previously inaccessible questions. For example, the lesion on the far right of Figure 4 is an encapsulated granuloma with characteristics fibrocaseous disease. Erosion of the capsule of such lesions was associated with reactivation TB in the lung.

**Figure 5:**
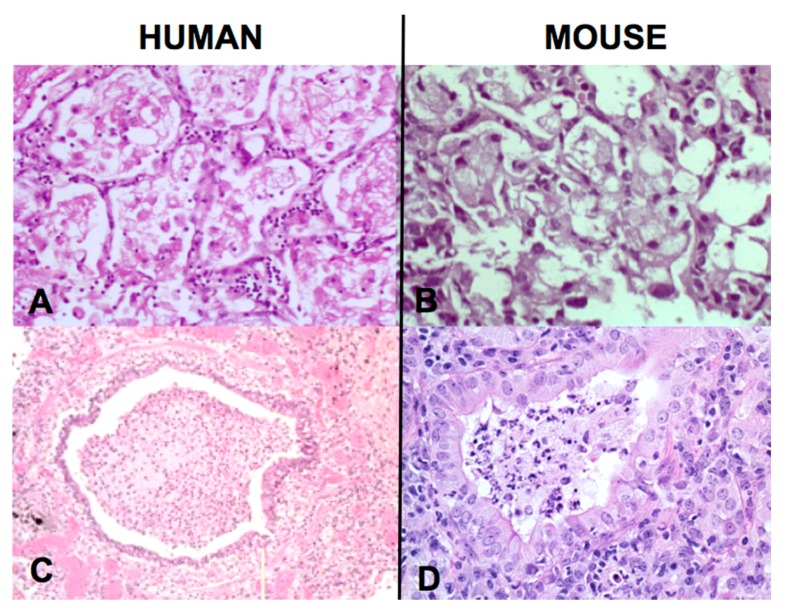
Comparison of early post-primary TB in humans with slowly progressive TB in mice [32]. Both lesions are obstructive lobular pneumonias characterized by foamy alveolar macrophages with very few AFB (**A**,**B**) (H&E stain 100×) and bronchial obstruction (**C**,**D**) (32) (H&E stain **A**, **B**, **C** 100×, **D** 200×).

**Figure 6:**
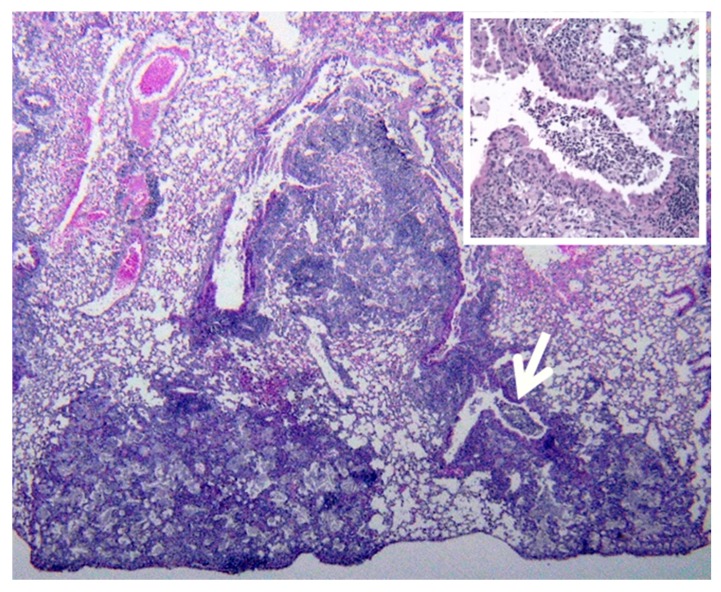
Reactivation Tuberculosis in the Mouse at 200 days. The Cornell model is produced by inducing latent TB in mice by treatment with antibiotics. The disease then reactivates months later as typical bronchogenic TB. It is a tuberculous lipid pneumonia quite different from the granulomas of primary murine TB. Like human post primary TB, it typically begins as sub pleural wedge shaped pneumonic lesions. Bronchial obstructions are frequently found at the apex of the wedge (arrow and insert). Acid fast bacilli are present in low numbers in alveolar macrophages. No granulomas are present in these animals. The dark purple areas are primarily interstitial lymphocytes while the pale areas they surround are alveoli filled with foamy macrophages. (H&E 100×).

Another example of value of human tissues for identifying animal models of particular human lesions is provided by slowly progressive TB in mice [37]. In humans, post-primary TB develops as bronchogenic spread of asymptomatic obstructive lobular pneumonia that has many similarities to slowly progressive TB in mice [32], Figure 5. The murine lesion is also a bronchogenic spread of obstructive lobular pneumonia that begins only after establishment of CMI with clearance of most AFB from granulomas in the lung, liver and spleen. It is characterized by lipid rich, foamy alveolar macrophages that accumulate mycobacterial antigens over time with few detectable AFB. These lesions expand until the animals rapidly develop symptoms and die due to pulmonary failure. Reactivation TB in mice produced by the Cornell model is an even better model of developing human post-primary TB because it begins like the human disease as sub pleura, wedge shaped lesions of bronchogenic obstructive lipid pneumonia, Figure 6.

**Figure 7:**
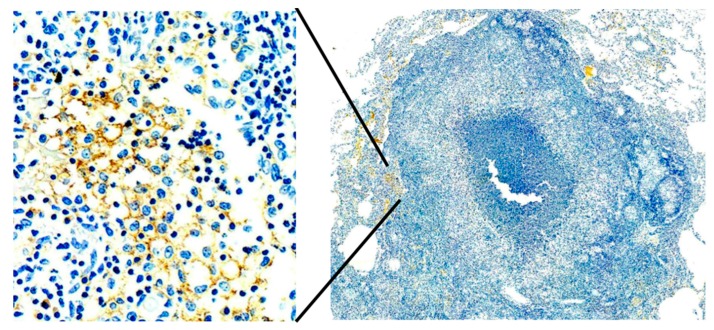
MTB antigen in a Macaque lung at 4 months after infection. MTB antigen (brown IHC stain) is present alveolar macrophages just outside of a granuloma. Earlier time points showed MTB antigen only in granulomas. This pattern of MTB antigen in alveolar macrophages outside of granulomas is typical of the earliest stage of post-primary TB in humans and mice. (IHC stain for MTB at 400× **left** and 40× **right**).

**Primates** are considered to be essential models of TB for both scientific and regulatory reasons. However, most studies have been driven by the paradigm that granulomas of primary TB are the key lesion of all TB. Bronchogenic TB with formation of cavities by dissolution of caseous pneumonia and post-primary granulomas that develop around preexisting caseous pneumonia have not been described in primates [33]. We believe that bronchogenic TB has not been described because it has not been a priority of investigation. There are descriptions of TB pneumonia, but they appear to be perifocal reactions to primary TB [38]. Since, bronchogenic TB develops late in sensitized animals, many studies have been of too short duration with inappropriate protocols for this purpose. Nevertheless, we have immunohistochemical (IHC) evidence suggesting that primates can develop early post-primary TB, Figure 7. In addition, the characteristic appearance of developing post-primary TB on high resolution CT scans, the tree-in-bud sign, has been reported in primates [39]. Finally, around 50% of tuberculous Rhesus Macaques develop bronchial obstruction due to enlarged lymph nodes. This happens in people and causes a post obstructive TB pneumonia that resolves spontaneously when the obstruction abates. This is called epituberculosis and is probably an excellent model of the earliest stage of post-primary TB [28].

## 2. Summary and Future Directions

MTB is more than a pathogenic bacteria. It is an obligate human parasite. Consequently, its interactions within humans are more complex than typical bacterial pathogens that do not depend upon infecting humans for survival. MTB, like malaria parasites, has multiple stages and disease processes within humans. MTB has two distinct stages, three if you count latency. The two stages, primary and post-primary TB differ from each other in the genetics host of predisposition, clinical presentation, age of onset, organ distribution of lesions, amount of necrosis and pathology [3,15,40]. All of the animal models are unnatural hosts in which MTB can not complete its life cycle. They all begin with granulomas resembling primary TB, but none of them are able to produce the lesions of post primary TB that mediate most clinical disease and nearly all transmission to new hosts.

Primary TB is the process of developing systemic immunity that protects the body from disseminated infection. Caseating granulomas are the characteristic lesion. It begins most often in the lung, but typically spreads to lymph nodes and many other organs. Primary TB disease occurs when systemic immunity is insufficient to control the organisms within granulomas either because of immunosuppression or inadequacy of prior immunization. In immunocompetent people, it heals in weeks with development of systemic immunity mediated largely by activated macrophages and granulomas. Immunization with BCG is effective in protecting against dissemination of primary TB.

Post-primary TB, also known as bronchogenic TB, always follows primary TB due to either reactivation of latent organisms or new infection from the environment. It occurs only in lungs, is characterized by extensive necrosis, produces most clinical disease and nearly al transmission of infection to new hosts. Post-primary TB begins as a silent prolonged local manipulation of host immunity in parts of the lung to produce conditions for sudden onset of caseous pneumonia [13,41]. There are no granulomas at this stage. It is an obstructive lobular pneumonia. The process may be aborted at multiple stages making completion to cavity formation a rare event. Acute post-primary disease results when the lesions undergo necrosis to produce caseous pneumonia that is either coughed out to leave a cavity or persists as fibrocaseous disease. Granulomas of chronic post-primary disease occur when necrotic caseous pneumonia is retained in the lung to become surrounded by fibrocaseous granulomas. BCG has little, or likely, no protective effect on post-primary TB. Evidence indicates that BCG simulates primary TB which is necessary for initiation of post-primary disease.

The prevailing paradigm that primary granulomas are the key lesion of both primary and post-primary TB is an oversimplification of the disease processes and impediment to understanding the biology of post-primary TB. The genesis of this situation is understandable. Post-primary TB has remained out of reach of most scientists for over 50 years. Autopsies on patients with untreated TB are now exceedingly rare in the developed world and the few people with access to such tissues are typically forensic pathologists have little interaction with scientists interested in TB. Furthermore, until recently, morphologic pathology had little new to contribute. This has now changed. Formalin fixed paraffin embedded (FPPE) blocks preserve the actual human disease with the microenvironment and regulatory molecules intact. Technologies to investigate such tissues have become quite sophisticated. We can determine the DNA sequence and gene expression of MTB in formalin fixed paraffin embedded (FPPE) blocks if human lungs. We can also conduct 8 color IHC studies on FPPE tissues with intact morphology allowing the characterization of multiple markers on many types of individual cells in their native environment [42]. Individual human lungs frequently contain both the early and late stages of post -primary disease so that it is possible to study much of the entire progression of disease in a single specimen. Animals models have contributed greatly to our understanding of disease mechanisms, cells, regulatory mechanisms and pathways, but with an oversimplified understanding of the lesions of developing post-primary TB, they have not been able to effectively address key questions. It seems that animal models are necessary to define the parts, but human tissues are needed to put them together correctly.

As stated by Robert North, a central problem in TB research is to explain why immunity to infection does not enable mice, guinea pigs, rabbits, or susceptible humans to resolve lung infection and thereby stop the development of disease [43]. This refers to the observation that the bacterial counts in the lungs of mice, guinea pigs, rabbits and susceptible humans all peak after a few weeks and then decline to a lower level where they remain stable for months in spite of systemic immunity. The animals, like some humans, then develop clinical disease and may succumb with little or no increase in the numbers of organisms [31]. This is the characteristic pattern of human post-primary TB that is reproduced in animal models. Understanding this assigns potential value to each of the commonly used animals for modeling specific components of the processes that occur in human tuberculosis.

Knowledge of the pathology of human TB makes it possible to manipulate animals to produce models of particular lesions. It appears that many mammals have the basic mechanisms of human TB, but fail to coordinate them as humans. Bronchoscopic infection of sensitized rabbits produces models of developing post-primary TB. Slowly progressive TB in immunocompetent mice is a model of the early stages of bronchogenic spread of obstructive lobular pneumonia of post-primary TB. We demonstrated that mice can produce typical caseating granulomas by reproducing in them the conditions that occur in humans at that stage of infection [35]. We are optimistic that coordinated study of human tuberculous lung tissue with such models can be used with modern technologies to finally address long-standing questions about host/parasite relationships in TB, and also be useful in developing targeted therapeutics and vaccines to combat this pathogen.

## References

[1] Huang L., Russell D.G. (2017). Protective immunity against tuberculosis: What does it look like and how do we find it?. Curr. Opin. Immunol..

[2] (2011). An International Roadmap for Tuberculosis Research: Towards a World Free of Tuberculosis.

[3] Hunter R.L. (2011). Pathology of post primary tuberculosis of the lung: An illustrated critical review. Tuberculosis (Edinb).

[4] Pai M., Behr M.A., Dowdy D., Dheda K., Divangahi M., Boehme C.C., Ginsberg A., Swaminathan S., Spigelman M., Getahun H. (2016). Tuberculosis. Nat. Rev. Dis. Primers.

[5] Dannenberg A.M. (2006). Pathogenisis of Human Pulmonary Tuberculosis. Insights from the Rabbit Model.

[6] Canetti G. (1968). Biology of the mycobacterioses. Pathogenesis of tuberculosis in man. Ann. N. Y. Acad Sci..

[7] Canetti G. (1955). The Tubercle Bacillus in the Pulmonary Lesion of Man. Histobacteriology and its Bearing on the Therapy of Pulmonary Tuberculosis.

[8] Laennec R. (1982). A Treatise on Diseases of the Chest in Which They are Described According to Their Anatomical Characters, and Their Diagnosis Established on a New Principle by Means of Acoustick Instruments.

[9] Bennett J.H. (1854). The Pathology and Treatment of Pulmonary Tuberculosis.

[10] Powell R.D. (1876). Chapter II. Pathology. On Consumption and on Certin Diseases of Lungs and Pleura.

[11] Cornil V., Ranvier L. (1880). Pathological Histology of the Respiratory Apparatus. A Manual of Pathological Histology Translated with Notes and Additions by EO Shakespeare and JHC Simms.

[12] Osler W., McCrae T. (1921). Chapter XXI, Tuberculosis. The Principles and Practice of Medicine.

[13] Opie E.L. (1927). Pathology of the Tuberculosis of Childhood and Its Bearing on Clinical Work. Br. Med. J..

[14] Kayne G.G., Pagel W., O’Shaughenessy L. (1939). Pulmonary Tuberculosis, Pathology, Diagnosis and Management.

[15] Rich A. (1951). The Pathogenesis of Tuberculosis.

[16] Medlar E.M. (1955). The behavior of pulmonary tuberculous lesions; a pathological study. Am. Rev. Tuberc..

[17] Pagel W., Simmonds F., MacDonald N., Nassau E. (1964). The Morbid Anatomy and Histology of Tuberculosis, an Introduction in Simple Terms. Pulmonary Tuberculosis, Bacteriology, Pathology, Management, Epidemiology and Prevention.

[18] Im J.G., Itoh H., Shim Y.S., Lee J.H., Ahn J., Han M.C., Noma S. (1993). Pulmonary tuberculosis: CT findings--early active disease and sequential change with antituberculous therapy. Radiology.

[19] Skoura E., Zumla A., Bomanji J. (2015). Official publication of the International Society for Infectious Diseases. Int. J. Infect. Dis..

[20] Dorhoi A., Kaufmann S.H. (2016). Pathology and immune reactivity: Understanding multidimensionality in pulmonary tuberculosis. Semin. Immunopathol..

[21] Subbian S., Tsenova L., Kim M.J., Wainwright H.C., Visser A., Bandyopadhyay N., Bader J.S., Karakousis P.C., Murrmann G.B., Bekker L.G. (2015). Lesion-Specific Immune Response in Granulomas of Patients with Pulmonary Tuberculosis: A Pilot Study. PLoS ONE.

[22] Hunter R.L. (2016). Tuberculosis as a three-act play: A new paradigm for the pathogenesis of pulmonary tuberculosis. Tuberculosis (Edinb).

[23] Nunes-Alves C., Booty M.G., Carpenter S.M., Jayaraman P., Rothchild A.C., Behar S.M. (2014). In search of a new paradigm for protective immunity to TB. Nat. Rev. Microbiol..

[24] Leong F.J., Dartois V., Dick T. (2011). A Color. Atlas of Comparative Pathology of Pulmonary Tuberculosis.

[25] Verma N., Chung J.H., Mohammed T.L. (2012). “Tree-in-bud sign”. J. Thorac. Imaging.

[26] Hunter R.L. (2011). On the pathogenesis of post primary tuberculosis: The role of bronchial obstruction in the pathogenesis of cavities. Tuberculosis (Edinb).

[27] Snider G.L., Radner D.B. (1955). Obstructive emphysema in pneumonia simulating cavity. Dis. Chest..

[28] Hutchison J.H. (1949). The pathogenesis of epituberculosis in children with a note on obstructive emphysema. Glasg. Med. J..

[29] Mustafa T., Leversen N.A., Sviland L., Wiker H.G. (2014). Differential in vivo expression of mycobacterial antigens in Mycobacterium tuberculosis infected lungs and lymph node tissues. BMC Infect. Dis..

[30] Youmans G.P., Youmans G.P. (1979). Pathogenesis of tuberculosis. Tuberculosis.

[31] Dannenberg A.M., Collins F.M. (2001). Progressive pulmonary tuberculosis is not due to increasing numbers of viable bacilli in rabbits, mice and guinea pigs, but is due to a continuous host response to mycobacterial products. Tuberculosis (Edinb).

[32] Hunter R.L., Jagannath C., Actor J.K. (2007). Pathology of postprimary tuberculosis in humans and mice: Contradiction of long-held beliefs. Tuberculosis (Edinb).

[33] Hunter R. (2016). Primate models of tuberculosis. Faith-based or evidence-based science. Tuberculosis (Edinb).

[34] Kubler A., Luna B., Larsson C., Ammerman N.C., Andrade B.B., Orandle M., Bock K.W., Xu Z., Bagci U., Molura D.J. (2015). Mycobacterium tuberculosis dysregulates MMP/TIMP balance to drive rapid cavitation and unrestrained bacterial proliferation. J. Pathol..

[35] Hunter R.L., Olsen M., Jagannath C., Actor J.K. (2006). Trehalose 6,6′-dimycolate and lipid in the pathogenesis of caseating granulomas of tuberculosis in mice. Am. J. Pathol..

[36] Dvorak H.F., Galli S.J., Dvorak A.M. (1986). Cellular and vascular manifestations of cell-mediated immunity. Hum. Pathol..

[37] Mustafa T., Phyu S., Nilsen R., Jonsson R., Bjune G. (1999). A mouse model for slowly progressive primary tuberculosis. Scand. J. Immunol..

[38] Lin P.L., Rodgers M., Smith L., Bigbee M., Myers A., Bigbee C., Chiosea I., Capuano S.V., Fuhrman C., Klein E. (2009). Quantitative comparison of active and latent tuberculosis in the cynomolgus macaque model. Infect. Immun..

[39] Sharpe S., White A., Gleeson F., McIntyre A., Smyth D., Clark S., Sarfas C., Laddy D., Rayner E., Hall G. (2016). Ultra low dose aerosol challenge with Mycobacterium tuberculosis leads to divergent outcomes in rhesus and cynomolgus macaques. Tuberculosis (Edinb).

[40] Alcais A., Fieschi C., Abel L., Casanova J.L. (2005). Tuberculosis in children and adults: Two distinct genetic diseases. J. Exp. Med..

[41] Kayne G.G. (1941). Origin, Diagnosis, and Management of Early Bronchogenic Tuberculosis. Br. Med. J..

[42] Blom S., Paavolainen L., Bychkov D., Turkki R., Maki-Teeri P., Hemmes A., Valimaki K., Lundin J., Kallioniemi O., Pellinen T. (2017). Systems pathology by multiplexed immunohistochemistry and whole-slide digital image analysis. Sci. Rep..

[43] North R.J., Jung Y.J. (2004). Immunity to tuberculosis. Annu. Rev. Immunol..

